# Gender-Specific Aspects of Teachers Regarding Working Behavior and Early Retirement

**DOI:** 10.3389/fpsyg.2022.829333

**Published:** 2022-02-15

**Authors:** Steffi Kreuzfeld, Reingard Seibt

**Affiliations:** Institute for Preventive Medicine, Rostock University Medical Center, University of Rostock, Rostock, Germany

**Keywords:** teachers, gender, overcommitment, recovery, retirement

## Abstract

Worldwide, a significant proportion of teachers retires prematurely for health reasons or at their own request. The study examines whether male and female teachers differ in terms of working conditions and coping with high work demands as well as individual factors that promote early retirement. A cross-sectional study was conducted to collect data from 6,109 full-time teachers in high schools (56% women). Weekly working hours from a four-week working time record and psychosocial work stress (effort-reward model, ER ratio) were used as workloads. In addition, emotional exhaustion (Maslach Burnout Inventory) and coping strategies that endangered health were recorded in the form of overcommitment and inability to recover. Also, the teachers gave a prediction and reasons for early retirement and made their own suggestions on how to prevent this. The results show that both workloads and emotional exhaustion are comparable between the genders, but women have a greater tendency than men to overcommit and be unable to recover. As ER ratio and emotional exhaustion increase, the chances for both genders to reach the regular retirement age decrease significantly; for health-endangering coping strategies, the relationship is somewhat weaker. The majority of male and female teachers (79%) indicates excessive workloads as the main reason for leaving the profession early. In order to protect teachers from high workloads, measures at the organizational, social, and individual level are necessary. Proposals for schools and policy makers are critically discussed on the basis of teacher recommendations.

## Introduction

The teaching profession is characterized by a complex structure of work demands and stressors. In addition to high mental, emotional, and psychosocial work load (Shirom et al., 2009; Skaalvik and Skaalvik, 2017a; Framke et al., 2021), the profession can be described by a high degree of autonomy. Compared to other occupational groups, there is an increased risk of stress-related psychosomatic and mental illnesses, including burnout (Guglielmi and Tatrow, 1998; García-Carmona et al., 2019). On the other hand, teachers are often more satisfied with their jobs than other professional groups despite the high demands (Schult et al., 2014).

With regard to work organization, the activities of the teachers show typical characteristics of the flexible working world with individualized working hours and locations. This goes hand in hand with a high level of personal responsibility for the results of the work, the risk of permeable boundaries between job and private life (Ashforth et al., 2000; Clark, 2000) and the risk of time and performance pressure hazardous to health (Höge and Hornung, 2013). However, many teachers appreciate the high degree of autonomy in their profession. In addition to a fixed number of teaching hours and regular extracurricular appointments (e.g., meetings), they are relatively free to allocate more than half of their working time (European Commission/Eurydice, 2013; OECD, 2019). However, the prerequisite for successfully coping with the extremely diverse work tasks is professional self-organization through which professional time and expenditure are individually controlled. In this respect, the subjective potential and resources of a teacher (e.g., work organization and ability to recover) are of considerable importance for the long-term preservation of health and work ability.

A conflict between self-determination and self-endangerment results from weighing up of one’s own quality standards at work and the need to recover from work. High expectations placed on teachers by society, parents, and students and their own desires for success, e.g., good student performance, can cause excessive exertion even though they realize that they are putting their health at risk (Dettmers et al., 2016). Krause et al. (2015) coined the term “interested self-endangerment” to describe this behavior. Typical examples of self-endangering behavior include presentism, working excessively long hours, working on weekends and vacations, and not taking recovery breaks. In a recent article, the working group around Krause demonstrated that teachers achieve short-term success in coping with their work demands by extending their working hours and thus experience themselves as competent. However, in the long term, they increase the risk of mental health impairments if they ignore the recovery required (Baeriswyl et al., 2021).

One model that analyzes the impact of job demands on teacher health is the effort-reward (ER) model (Siegrist et al., 2009). High effort is caused by working under time pressure, working with interruptions, or by an increase in workload. Reward, on the contrary, subsumes both material aspects, such as salary, promotion opportunities, and job security, and immaterial aspects, such as appreciation by colleagues, superiors, students, and parents. In a favorable case, for example, teachers’ high workload is compensated by adequate pay and appreciation by colleagues and students. According to this model, an effort-reward imbalance (ERI) generates psychosocial work stress that increases the risk of stress-associated illness and burnout in the medium and long term (Van Vegchel et al., 2005, Lehr et al., 2009; Zurlo et al., 2010; Wang et al., 2015; Rugulies et al., 2017; Solis-Soto et al., 2019; Madsen and Rugulies, 2021). In the study by Loerbroks et al. (2014), the ERI score in elementary school teachers was found to be a strong determinant not only of burnout but also of intention to leave the profession. Niedhammer et al. (2014) postulate that 16% of mental disorders can be attributed to an ERI.

In addition to work stress (extrinsic factor), the ER model also includes overcommitment (OC) as an intrinsic component. This describes an individual coping style with the tendency to overexert oneself without regard to one’s own resources (Siegrist and Li, 2016). It assumes that teachers who are simultaneously characterized by high ERI and high OC are at the greatest risk of decreased health and wellbeing (Siegrist et al., 2009; Siegrist and Li, 2016; Hinsch et al., 2019). However, the evidence on this is inconsistent. Excessive work engagement can, on the one hand, lead to extended working hours and, on the other hand, have unfavorable effects on recovery processes during non-working time, e.g., by not fully compensating for the consequences of previous activities (Meijman and Mulder, 1998; Sonnentag, 2003). Both long working hours and shortened recovery times are relevant to health (Meijman and Mulder, 1998; Wepfer et al., 2018).

Teachers working full-time in Germany often have high weekly working hours (Ø 45 h/week; Felsing et al., 2019a). Many even have to regularly use the weekend to manage their workload. In doing so, longer recovery intervals are fragmented. As a result, teachers have more limited recovery opportunities than people in other occupations with shorter working weeks or part-time work.

Recovery processes may also be impaired in teachers if they are unable to sufficiently distance themselves from work-related content (Sonnentag and Fritz, 2015). This mental detachment from work during rest periods is seen as a central component of individual recovery (Sonnentag and Fritz, 2015; Stieler et al., 2019). It is considered a link between working conditions and stress-related outcomes and has been discussed as an early indicator of exhaustion and burnout (Wendsche and Lohmann-Haislah, 2017; Seibt and Kreuzfeld, 2021). A physiological activation which lasts for the duration of working time is seen as a pathomechanism (Sonnentag and Fritz, 2015) which hinders the necessary recovery associated with persistent cognitive processes, such as affective rumination (Rau and Triemer, 2004; Cropley and Zijlstra, 2011). Also, excessive work engagement can thus contribute to individuals losing the ability to relax. This inability to recover is considered an individual pattern in coping with work demands and is considered a health risk factor in its own right among teachers (Varol et al., 2021). In a longitudinal study, Sonnentag et al. (2010) were able to predict future exhaustion for employees who have poor mental detachment in their free time. Exhaustion in this respect is the result of a chronic overtaxing of one’s own performance reserves. In the “burnout concept” according to Maslach and Jackson (1981), emotional exhaustion is seen as the core component (Hakanen et al., 2006; Skaalvik and Skaalvik, 2011a).

Gender seems to play an important role in relation to the extent of work stress. However, previous studies have provided contradictory findings in this regard (Gyllensten and Palmer, 2005). Women and men both differ in the way they are exposed to stress and in their response to stress (Folkman et al., 1986; Arntén et al., 2008). Causal factors include differences in working conditions, social role behavior, role conflicts, especially work-family conflicts, gender stereotypes, and related differences in advancement opportunities, among others (Gyllensten and Palmer, 2005; Li et al., 2006). In principle, workplace stress and work-family conflicts are risk factors for mental health disorders in both genders (Wang et al., 2008). For women, however, the probability of work-family conflicts and emotional exhaustion increases when they have to work longer than desired, i.e., they suffer from over-employment (Rubino et al., 2013).

Research has considered the differential effects of stress on men’s and women’s health under two hypotheses: differential exposure and differential vulnerability. The first hypothesis assumes that with fewer work-related resources (e.g., income and job promotion), women are exposed to interpersonal, emotional, and social stressors, including work-family conflicts, to a greater extent than men and therefore complain more about stress-related problems (e.g., Bond et al., 2004). The second hypothesis assumes that women react more sensitively to certain stressors because of the additive effect of family and paid work roles (Roxburgh, 1996; Arroba and James, 2002; Liu et al., 2008). It is conceivable that the observed gender differences will be reduced by the convergence of social roles (e.g., fathers taking on more family responsibilities) and the equalization of working conditions (Frankenhaeuser, 1991; Persson et al., 2008).

Decades ago, there were some studies that found gender differences in stress triggers and perceptions of stress levels among teachers as well (Laughlin, 1984; Travers and Cooper, 1991). In contrast, other studies found no gender-based differences (Jepson and Forrest, 2006; Reilly et al., 2014). Subsequently, the most common causes of occupational stress among teachers were identified as high work demands, student misbehavior, lack of student interest and motivation, and difficult interactions with colleagues and parents (Borg, 1990; Klassen et al., 2012; Aldrup et al., 2018). Here, female teachers have reported significantly higher levels of occupational stress than their male colleagues particularly in interactions with students and colleagues (Griffith et al., 1999; Antoniou et al., 2006). Females also reported higher levels of workload and emotional exhaustion compared to their male counterparts (Van Dick and Wagner, 2001; Sünbül, 2003; Antoniou et al., 2006; Wang et al., 2015; Arvidsson et al., 2016), and more discomfort and a higher anxiety level (Tamres et al., 2002; Chong and Chan, 2010; Arvidsson et al., 2016). Overall, female teachers perceive stress more often (Greenglass and Burke, 2003; Rasku and Kinnunen, 2003; Antoniou et al., 2006; Chaplain, 2008; Agaiâ-Demjaha et al., 2015) and rate their health worse than their male colleagues (Lagrosen and Lagrosen, 2020). In addition to gender, the number of working hours and relationship status are further factors influencing the extent of emotional exhaustion among teachers. Thus, married and partnered teachers as well as teachers with a weekly working time of less than 40 h each reported a lower emotional exhaustion than singles and employees with a weekly working time of more than 40 h (Wang et al., 2015).

Some teachers only become aware of the finite nature of their own resources when they are emotionally exhausted or suffer burnout. Latest now, there is a real risk of them having to give up the teaching profession and taking early retirement. Mental illnesses, especially emotional exhaustion, are closely related to early retirement among teachers (Leung and Lee, 2006; Skaalvik and Skaalvik, 2011b). Early retirement is understood here as the time of complete withdrawal from the teaching profession before reaching the official retirement age. In research, retirement is viewed as a process that examines retirement planning and the decision to retire, as well as retirement with its corresponding consequences (Beehr, 1986; Topa et al., 2009; Fisher et al., 2016a; Topa et al., 2018). This very complex process is influenced by a variety of individual, family, and work-related factors. In this context, personal goals interact with financial and health constraints.

In Germany, the regular retirement age for teachers is 67 years. However, only about one in four teachers reaches the statutory retirement age (Statistisches Bundesamt, 2018). Concrete data on the proportion of teachers who retire early due to invalidity are not available for Germany. A significant proportion of teachers still leave the profession early at their own request. According to research by Van Droogenbroeck and Spruyt (2014), female teachers are more likely than male teachers to want to retire.

In summary, it can be said that working conditions as well as individual and health-related factors can promote early retirement among teachers. With regard to previous studies on work-related stress among teachers, it should be critically noted that working conditions were often the focus of studies and that very heterogeneous samples are examined, including both full-time and part-time employees as well as teachers from primary and high schools, and teachers with different job profiles (e.g., principals, teachers with special functions, and regular teachers). This severely limits the interpretability of the results and may lead to incorrect conclusions. Therefore, further studies with homogeneous teacher samples are needed to analyze the causes of gender differences in work-related stress outcomes, paying particular attention to individual factors.

The aim of the study was therefore to identify possible gender differences in work, personal, and health characteristics on the basis of a dataset that is representative for full-time high school teachers in Germany. In particular, aspects of self-harming behavior were to be considered. Furthermore, it was necessary to clarify the question of the predictive value individual characteristics have for the probability of reaching regular retirement age. In addition, the analysis covered whether the subjective reasons for early retirement differed between female and male teachers.

## Materials and Methods

### Procedures and Data Collection

The data for the present study were collected as part of the Germany-wide, cross-sectional study “Lehrerarbeit im Wandel” (Teaching under Change - LaiW study) between January and April 2018. The study determined the workload and health of high school (in German: Gymnasium) teachers in all 16 German states. The study period selected for the individual federal state represented an average workload in each case (no extraordinary activities, such as exams or extensive correction work).

In the run-up to the study, posters and flyers were placed at all high schools to advertise voluntary participation. Before the start of the study, all teachers received an information letter on the study in their school with information on data protection, implementation, and data evaluation as well as on the conditions for participation and access to the study. Anonymity of the data was ensured *via* transaction numbers and an eight-digit personal code. Data collection took place *via* an online portal of the University of Rostock.

A list of answers to frequently asked questions was available to participants on the LaiW study website for queries. In addition, the study team could be contacted by telephone and electronically throughout the study period.

The study consisted of an online questionnaire (OQ) and an online protocol (OP). First, all participants answered the online questionnaire on sociodemographic, job-specific, and health-related questions once. Subsequently, they logged their working time daily in the online protocol over a period of 4 weeks (28 days) using defined activity categories. From this, an average weekly working time was determined.

More than 20,000 high school teachers (hereafter teachers) participated in the LaiW study. Using the personal code, the online questionnaire and online protocol could be merged for data analysis. Complete datasets were available for 14,338 participants due to matching codes. About 84% of these records (*n* = 12,014) were related to teachers who primarily give lessons. In contrast, 16% of the records referred to teachers who were employed as head teachers or deputy head teachers or who performed other administrative tasks and functions within the school to a considerable extent and therefore gave significantly fewer lessons. For the comparison of gender-specific aspects, a sample should be studied that was as homogeneous as possible. Therefore, only datasets from full-time teachers with a reduction of up to 3 h (reduced teaching hours) were analyzed (*n* = 6,109).

### Sample

The sample of 6,109 full-time teachers was composed of a slightly higher proportion of women (56%) compared to men (44%). The mean age of men was 42 ± 10 years and that of women was 41 ± 10 years (*d* = 0.180). The further composition of the sample is summarized in Table 1. Please see data analyses for information on the interpretation of effect sizes (d).

**Table 1:** Characteristics of the sample of male and female full-time teachers.

	Full-time teacher				Significance	
	Male (*n* = 2,680)		Female (*n* = 3,429)		Test value	Value of *p* (effect size)
	%	*n*	%	*n*		
**Age groups [years]**	5.1	138	14.5	498		
25–29	39.9	1,069	40.0	1,373	215.51	<0.001 (0.382)
30–39	31.3	840	20.3	695		
40–49	17.5	469	20.6	708		
50–59	6.1	164	4.5	155		
60–67	5.1	138	14.5	498		
**Subjects and subject combinations**						
Languages	7.9	213	22.7	777	398.95	<0.001 (0.529)
Social sciences	5.6	149	2.1	71		
Natural sciences	27.5	736	16.7	574		
Languages and social sciences	23.8	638	25.1	859		
Languages and natural sciences	3.7	99	8.0	274		
Social sciences and natural sciences	8.6	230	7.0	241		
Art, music, sports	2.5	66	1.7	60		
Subject combinations with art, music, sports	20.5	549	16.7	573		
**Family obligations**						
Permanent partnership	86.7	2,323	76.8	2,635	95.15	<0.001 (0.252)
Children in the household	52.0	1,394	21.4	733	699.16	<0.001 (0.719)
Care of relatives	4.5	120	5.8	198	5.13	0.024 (0.058)

%: frequencies in %; *n*: number of teachers. Chi-square test according to Pearson (test size: *χ*^2^-value, effect size: *d*); p-value: significance (two-sided). Effect size according to Cohen (1988): *d*: <0.20 = no effect, 0.20–0.49 = small effect, 0.50–0.79 = medium effect, and ≥0.80 = large effect.

Combinations of languages and social sciences as well as languages and natural sciences were taught most frequently, with women indicating more pure language subjects and men indicating more natural science subjects (*d* = 0.529, medium effect).

Most teachers lived in a stable partnership (men: 87%, women: 77%; *d* = 0.252, small effect). About 5% of teachers reported having to care for relatives in the household, and more than a third (35%) of them also took care of children in their own household; however, this applied to 52% of men but only 21% of women (*d* = 0.719, medium effect).

### Measures

Working time and the characteristics of psychosocial workload are used to describe workload (Siegrist et al., 2009). Overcommitment (Siegrist et al., 2009) and inability to recover (Richter et al., 1996) are attributes of self-harming behavior and emotional exhaustion is considered a health-related characteristic (Schaufeli et al., 1996). All questions about future retirement were developed in-house.

### Online Questionnaire

In addition to sociodemographic (e.g., gender, age, and marital status) and occupation-specific information about teachers (e.g., teaching responsibilities, subjects taught, classes, and number of students), the OQ also included questions about work, personal, and health characteristics. Standardized questionnaires and supplementary self-developed questions were used to record these characteristics.

#### Psychosocial Workload

It was surveyed with the Effort-Reward-Imbalance Questionnaire (ERI-Q: Siegrist et al., 2009). This questionnaire allows the standardized measurement of occupational gratification crises. The short version used by Siegrist et al. (2009) included the main scales effort (3 items; range: 3–15 points) and reward (7 items; range: 7–35 points), as well as the effort-reward ratio (ER ratio). Each effort item was measured on a five-point scale from 1 (“disagree”) to 5 (“agree, and I’m very distressed”). The reward scale consisted of three subscales: status or job promotion, valuation or esteem, and job security. The reward items were measured on a five-point scale from 1 (“agree) to 5 (“disagree, and I’m very distressed”). High sum values indicated high perceived effort or reward. The ER ratio was formed from the sum values of the two main subscales by the following rule: ER-Ratio *=* ∑ effort/(∑ reward ^*^ 0.54). An ER ratio of >1 indicates an ERI (Siegrist et al., 2009), which is said to be associated with a health risk. The greater the imbalance between effort and reward (gratification crisis), the higher the health risk is said to be. Validity and reliability of the German short version of ERI-Q were satisfactory (Siegrist et al., 2009). For the main subscales, the values of internal consistency were above 0.70 (effort: 0.74 and reward: 0.79). For the ER scales of the present study, lower Cronbach Alpha’s were determined (effort: 0.61 and reward: 0.72), which can be classified as questionable or acceptable (Blanz, 2015).

#### Overcommitment

Overcommitment (OC) or excessive work commitment was also assessed with the short version of the ERI-Q (Siegrist et al., 2009). This is an individual coping style with a tendency to spend oneself without regard to one’s resources. The OC scale comprises six items that are rated on a four-point Likert scale (1 = strongly disagree up to 4 = strongly agree). In this scale, a sum score is formed from the six items (value range: 6–24 points), in which high values correspond to a high propensity to exert oneself. The upper tercile of the sum score was defined as the risk group (Siegrist et al., 2004). A Cronbach’s Alpha of 0.79 is given as the internal consistency of the OC subscale (Siegrist et al., 2009). In the present LaiW study, the Cronbach’s Alpha for OC was 0.77, which is acceptable (Blanz, 2015).

#### Inability to Recover

Inability to recover (IR) is a subscale of Questionnaire for Faulty Attitudes and Behavior Analysis relevant to coping with work demands (Richter et al., 1996). Depicted is extreme work commitment associated with accepted limited recovery ability in terms of an inefficient coping style (Richter et al., 1999). The inability to recover is assessed with six items using a four-point ranking scale (1 = not at all true to 4 = very true). Then, the sum value (range: 6–24 points) is formed over the six items, which can be assigned on the basis of percentile values to normal (6–18 points), high (19–21 points), and very high (22–24 points) recovery values. The reliability of the IR subscale was reported by Richter et al. (2015) with a Cronbach’s Alpha of 0.79. The Cronbach’s Alpha for IR was calculated to be 0.82 in our study and can be assigned to the good range (Blanz, 2015).

#### Emotional Exhaustion

Emotional exhaustion (EE) is considered the core component of the frequently cited burnout definition of Maslach and Jackson (1981) and was recorded by the German translation of the Maslach Burnout Inventory - General Survey (MBI-GS: Schaufeli et al., 1996). The subscale EE consists of five statements (items), which are assessed on a seven-point Likert scale (0 = never up to 6 = daily) according to their frequency of occurrence and are summarized as a mean value to form the EE score. High EE scores indicate typical stress reactions and the draining of emotional resources. For evaluation, the mean values of the subscale EE can be classified as low (<2.0 points), average (2.0–3.2 points), and high (>3.2 points; Maslach and Jackson, 1986). The validity evidence of the MBI has been demonstrated both for normal and clinical populations (Schaufeli et al., 2001) and for different occupational groups. Maslach and Jackson (1986) presented internal consistencies in the form of Cronbach’s Alphas of 0.90 for emotional exhaustion for a sample of 1,316 subjects. Schaufeli et al. (1996) report a Cronbach’s Alpha of 0.78 for emotional exhaustion. For the study presented here, the Cronbach’s Alpha was 0.78, which is in the acceptable range according to Blanz (2015).

#### Time of Retirement

The questions about the probability of early versus regular retirement and about the individual reasons were developed in-house for pragmatic considerations and were each recorded with a global question. The following question was to be answered as: “Can you imagine practicing your profession until the statutory regular retirement age?” If the question was answered “no,” a maximum of three main reasons for early retirement was to be given. Similarly, participants were asked to suggest two to three specific actions that they believe were necessary to remain healthy and employed in the teaching profession until regular retirement age. These statements and the reasons for early retirement were free text statements that were manually evaluated or categorized for all 6,109 teachers.

### Online Protocol

The OP served to determine the weekly working time and activity structure of the teachers. To do this, the teachers had to document their work time daily for 4 weeks (28 days) using 12 practicable, suitably clear categories of teacher-specific activity, which were grouped into the following higher-level domains:

– teaching (lessons, substitution lessons)
– teaching-related activities (preparation and follow-up of lessons, correction and grading of students’ work, marking, preparation of projects, and excursions)
– non-teaching activities (work with students and parents, administration, work with colleagues, tasks within the scope of students’ inclusion and integration, supervision time, and all other tasks).

The total weekly working time was calculated by first determining the average values over 4 weeks for each activity category and subsequently summarizing these as the weekly working time. The amount of time for the individual activity categories was previously examined for statistical outliers. Extreme values were replaced with subject-specific mean values within each activity category. Participants who recorded their working time on fewer than 21 of 28 days were not included in the data analysis.

### Data Analyses

Prior to the statistical calculations, the entire dataset was checked for implausible data. Input aids and default settings in both the online questionnaire and the online working time log prevented implausible data from being entered.

Statistical analysis of the data was performed with the Statistical Package for the Social Science (SPSS INC, Chicago, IL, United States) for Windows (version 27). A probability of error of *α* < 0.05 was set as the statistical significance criterion and supplemented by effect sizes. The interpretation of effect sizes was based on the conventions of Cohen (1988). Statistically significant effects in the analyses of variance or the *χ*^2^ tests were considered to be small effect sizes from $\eta_{\mathrm{partial}}^{2}$ = 0.01 or *d* = 0.20, respectively.

The focus of this paper is on the analysis of gender effects for the examined work-, person-, and health-related characteristics. Mean differences between male and female teachers were examined for these characteristics - after checking for age groups and subject profiles - using univariate General Linear Models. The *χ*^2^ test was used for difference testing of categorical variables.

Correlations between work-related and personal characteristics and emotional exhaustion with the variable early or regular retirement were examined gender-specifically and with point-biserial correlations. Correlations between the characteristics were analyzed using Pearson product-moment correlation. Correlation coefficients were interpreted according to Bühl (2016), where *r* ± ≤0.10 was considered independent of each other.

Binary logistic regression analyses were carried out to clarify the question of the predictive value work and individual characteristics (independent variables), including control variables, have for the probability of reaching the regular retirement age (response variables). These analyses were performed separately for male and female teachers. The selection of characteristics included in the overall model (method: enter) was based on the results of the correlation analysis; this was prefixed to the regression. To assess the goodness-of-fit, the Nagelkerke *R*^2^ was used, which can assume values of between 0 and 1.

## Results

### Gender Comparison for Workload

Weekly teaching hours, time for teaching-related and non-teaching activities, and working time were investigated as working time-related characteristics (see Table 2). As expected, the number of compulsory hours does not differ between male and female teachers (*p* = 0.234); they teach an average of 22 school hours per week (á 45 min). For teaching-related activities, however, women report an average of 19 h/week, about 2 h more than men ${\mathrm{(\eta}}_{\mathrm{partial}}^{2}$ = 0.016, small effect), while there is only a marginal gender effect for the time spent on non-teaching activities ${\mathrm{(\eta}}_{\mathrm{partial}}^{2}$ = 0.003); on average, all teachers invest 10 h/week for these tasks. In summary, female teachers work an average of 1.5 h more per week than male teachers (∅ 45.7 vs. 44.2 h/week; $\eta_{\mathrm{partial}}^{2}$ = 0.012, small effect).

**Table 2:** Main effects of work-related characteristics and covariates (age groups and subject profile) for male and female full-time teachers.

	Full-time teacher			Significance		
	Dimension	Male (*n* = 2,680)	Female (*n* = 3,429)	*F*-value	Value of *p*	Effect sizes ${\mathrm{(\eta}}_{partia\mathrm{l, d)}}^{2}$
Workload						
Teaching [hours/week, á 45 min]	M ± SD	22.5 ± 3.5	22.7 ± 3.5	1.42	0.234	0.001
Age group				62.16	<0.001^***^	0.009
Subject profile				0.17	0.680	0.001
Teaching-related activities [hours/week]	M ± SD	17.3 ± 6.6	19.4 ± 7.1	96.68	<0.001^***^	0.016
Age group				24.64	<0.001^***^	0.004
Subject profile				140.43	<0.001^***^	0.022
Non-teaching activities [hours/week]	M ± SD	10.0 ± 3.5	9.3 ± 3.1	20.94	<0.001^***^	0.003
Age group				57.69	<0.001^***^	0.009
Subject profile				7.93	<0.005^**^	0.001
Working time [hours/week]	M ± SD	44.2 ± 8.6	45.7 ± 8.7	31.93	<0.001^***^	0.005
Age group				43.94	<0.001^***^	0.007
Subject profile				13.22	<0.001^***^	0.002
Effort-reward subscales						
Effort [5–15 pts]	M ± SD	9.7 ± 2.6	9.5 ± 2.6	0.89	0.345	0.001
Age group				121.7	<0.001^***^	0.019
Subject profile				1.16	0.345	0.001
Reward [7–35 pts]	M ± SD	26.0 ± 5.4	26.0 ± 5.4	0.00	0.955	<0.001
Age group				20.45	<0.001^***^	0.003
Subject profile				8.22	0.004^**^	0.004
Effort-reward ratio (ER ratio)	M ± SD	0.93 ± 0.42	0.92 ± 0.43	0.19	0.665	<0.001
Age group				62.92	<0.001^***^	0.009
Subject profile				7.12	0.008^**^	0.001
Evaluation of ER ratio						
ER ratio ≤ 1	% (n)	64.3 (1,724)	66.1 (2,266)	2.04	0.153	0.037
ER ratio > 1	% (n)	35.7 (956)	33.9 (1,163)			

pts: points; M ± SD: mean ± standard deviation; % (n): frequency in %, n: number of teachers. Chi-square test according to Pearson (test size: *χ*^2^-value, effect size: *d*); univariate analyses of variance, design: constant term, sex + age group + subject profile (test size: *F*-value, effect size: $\eta_{\mathrm{partial}}^{2}$ : partial eta-square); value of *p*: significance (two-sided): ^***^p < 0.001, ^**^p < 0.01. Effect size according to Cohen (1988): $\eta_{\mathrm{partial}}^{2}$ : <0.01 = no effect, *d*: <0.20 = no effect. There are no age and subject profile effects for the effort-reward subscales ${\mathrm{(\eta}}_{\mathrm{partial}}^{2}$ <0.010). Corrected R-squared: teaching = 0.009, teaching-related activities = 0.044, non-teaching activities = 0.015, working time = 0.015, effort = 0.015, reward = 0.005, and effort-reward ratio = 0.010.

For the control variables age group and subject profile, there are statistically significant effects (*p* < 0.05) for activity proportions and working time, but it is not practically significant ${\mathrm{(\eta}}_{\mathrm{partial}}^{2}$ < 0.010). Younger colleagues (20–29 years) nevertheless have significantly longer working hours than older colleagues (60–67 years; ∅ 47.0 vs. 42.2 h/week; $\eta_{\mathrm{partial}}^{2}$ = 0.012, small effect). When looking at it in terms of gender, this difference can only be confirmed for female teachers (∅ 47.4 vs. 42.9 h/week; $\eta_{\mathrm{partial}}^{2}$ = 0.011, small effect).

The effort-reward subscales (ER subscales) are considered a second aspect of workload (see Table 2). The mean scores of the ER subscales are not significantly different between male and female teachers (*p* ≥ 0.05). The mean scores of all teachers are still in the normal range for effort (∅ 10 of 15 points) and reward (∅ 26 of 35 points). For the ER ratio, the mean value of the teachers is 0.93 and thus still outside the risk range. Nevertheless, there is a health risk due to the imbalance of effort and reward (ER ratio > 1) for more than one-third of them (35%). The three ER subscales effort, reward, and ER ratio are not influenced by the subject profiles taught ${\mathrm{(\eta}}_{\mathrm{partial}}^{2}$ < 0.010). However, there is an age effect for the results on effort ${\mathrm{(\eta}}_{\mathrm{partial}}^{2}$ = 0.019, small effect).

Neither gender differences nor age effects nor effects related to the subject profile can be determined with significant practical importance for the three reward subscales promotion, esteem, and job security ${\mathrm{(\eta}}_{\mathrm{partial}}^{2}$ < 0.010). The opportunities for job promotion are reported by the teachers with an average of 11 points (range: 3–15 points). Perceived professional esteem is rated an average of 7, and job security with 8 points (range in each case: 2–10 points).

### Gender Comparison for Personal Characteristics

Overcommitment and inability to recover were investigated as person-related characteristics with a link to self-harming behavior. On average, the mean scores for both overcommitment and inability to recover differ between male and female teachers (see Table 3; $\eta_{\mathrm{partial}}^{2}$ ≥ 0.01, small effects). These are within the normal range for both genders and both characteristics but are close to the border of the high range (>18 of 24 points).

**Table 3:** Main effects of personal characteristics and covariates (age groups and subject profile) of male and female full-time teachers.

Personal characteristics	Dimension	Male (*n* = 2,680)	Female (*n* = 3,429)	*F*-value	Value of *p*	Effect sizes ${\mathrm{(\eta}}_{partial}^{2}$ )
Overcommitment (OC) [6–24 pts]	M ± SD	16.8 ± 3.5	18.1 ± 3.3	207.51	<0.001^***^	0.033
Age group				4.14	0.042 ^*^	0.001
Subject profile				10.26	0.001^***^	0.002
Inability to recover (IR) [6–24 pts]	M ± SD	16.5 ± 3.6	17.7 ± 3.4	186.32	<0.001^***^	0.030
Age group				3.73	0.053	0.001
Subject profile				9.11	0.003^**^	0.001

pts: points; M ± SD: mean ± standard deviation; univariate analyses of variance, design: constant term + sex + age group + subject profile; test size: *F*-value, value of p: significance (two-sided): ^***^p < 0.001, ^**^p < 0.01, and ^*^p < 0.05. Effect size according to Cohen (1988): $\eta_{\mathrm{partial}}^{2}$ : <0.01 = no effect, 0.01–0.05 = small effect. Corrected R-squared: OC = 0.037, IR = 0.031.

**Figure 1:**
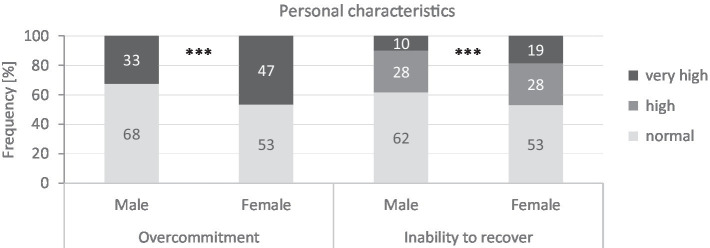
Overcommitment and inability to recover of male (*n* = 2,680) and female (*n* = 3,429) full-time teachers. Chi-square test according to Pearson (test size: *χ*^2^-value, effect size: d); significance (two-sided): ^***^p < 0.001. Effect size according to Cohen (1988): *d*: 0.20–0.49 = small effect.

One-third of male teachers (33%) and about half of female teachers (47%) tend to overexert themselves (see Figure 1, *d* = 0.288, small effect). A similar pattern emerges for inability to recover. Here too, significantly more female than male teachers show high or very high values (*d* = 0.250, small effect) at 47 and 38%, respectively. Age and subject profile have no relevant influence on these results ${\mathrm{(\eta}}_{\mathrm{partial}}^{2}$ < 0.010).

From the perspective of personal characteristics, there is a clear health risk from self-harming behavior for a total of 21% of male teachers and for more than one-third (35%) of female teachers; they are noticable for both high values for overcommitment and inability to recover. Only half of the teachers (51%) show normal levels of overcommitment and recovery at the same time (men: 59% and women: 44%).

### Gender Comparison for Emotional Exhaustion

There is a significant difference between male and female teachers (*p* < 0.001) for emotional exhaustion - checking for age groups and subject profile - but this difference is also not practically relevant ${\mathrm{(\eta}}_{\mathrm{partial}}^{2}$ < 0.01; see Table 4). Thus, the average values of teachers (range: 0–6 points) for emotional exhaustion are at 2.4 points. According to this, teachers experience emotional exhaustion on average “once a month.” This result is not influenced by age effects or effects of the subject profiles taught ${\mathrm{(\eta}}_{\mathrm{partial}}^{2}$ < 0.01).

**Table 4:** Main effects of emotional exhaustion and covariates (age groups and subject profile) of male and female full-time teachers.

	Full-time teacher	Significance			
	Male (*n* = 2,680)	Female (*n* = 3,429)	*F*-value	Value of *p*	Effect sizes ${\mathrm{(\eta}}_{partial}^{2}$ )
Corrected model			20.07	<0.001^***^	0.010
Constant term			2400.26	<0.001^***^	0.282
Emotional exhaustion	2.2 ± 1.3	2.5 ± 1.2	59.50	<0.001^***^	0.009
Age group			0.07	0.796	<0.001
Subject profile			0.07	0.788	<0.001

pts: points; means ± standard deviations; univariate analysis of variance, design: constant term, sex + age group + subject profile (test size: *F*-value (Fishers F), effect size: $\eta_{\mathrm{partial}}^{2}$ : partial eta-square); value of p: significance (two-sided): ^***^p < 0.001. Effect size according to Cohen (1988): $\eta_{\mathrm{partial}}^{2}$ : <0.01 = no effect, 0.01–0.05 = small effect, and ≥0.14 = large effect. Corrected R-squared = 0.009.

**Figure 2:**
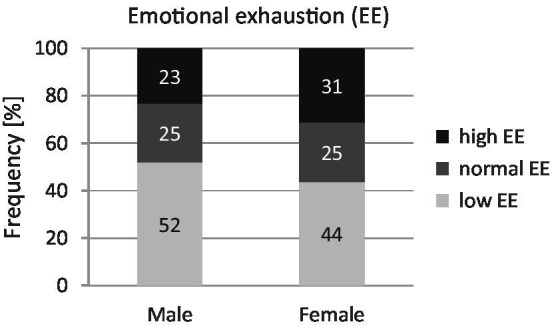
Emotional exhaustion of male (*n* = 2,680) and female (*n* = 3,429) full-time teachers. Chi-square test according to Pearson (test size: *χ*^2^-value, effect size: d); significance (two-sided). Effect size according to Cohen (1988): *d*: <0.20 = no effect.

According to the classification recommended by Maslach and Jackson (1986), the mean values of emotional exhaustion are in the average range for both genders. Just under a quarter (23%) of the male teachers and a third (31%) of the female teachers show high emotional exhaustion (see Figure 2).

### Gender Comparison for the Time of Retirement

**Figure 3:**
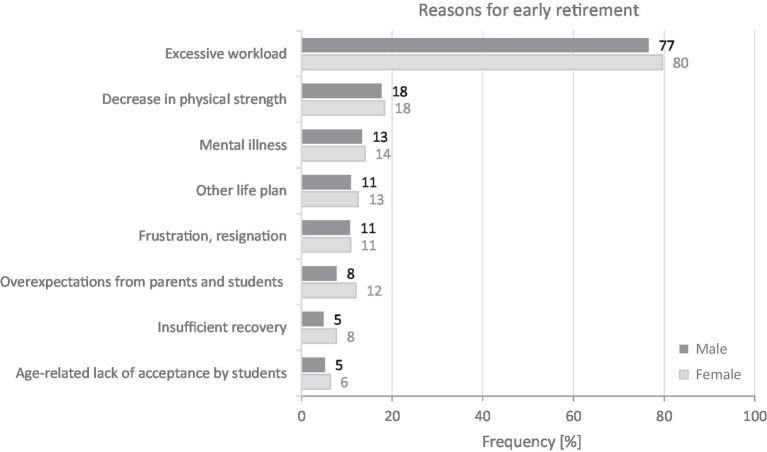
Reasons for not reaching regular retirement of male (*n* = 2,680) and female (*n* = 3,429) full-time teachers (multiple responses possible).

The question about early retirement was answered significantly differently by male and female teachers (*d* = 0.261, small effect): 30% of male and 42% of female teachers estimate that they will not remain in the profession until they reach the regular retirement age. The reasons for this hardly differ between the two gender groups (*d* < 0.20; see Figure 3). About half (51%) of the teachers made two statements and 13% made three. More than three quarters (79%) cited excessive workload as the main reason for taking early retirement. Age-related decrease in physical strength (18%) or mental illness (including emotional exhaustion; 18%) was also cited. For a few female teachers (<1%), caring for relatives is also a reason for early retirement. About 3% of teachers provided incorrect or no information on reasons for early retirement. Further reasons are shown in Figure 3.

In order to reach the regular retirement age in good health, teachers suggest the following main measures: reducing the number of compulsory hours (46%), decreasing class size (29%), reducing additional tasks (21%) as well as bureaucracy and the administrative burden (18%), and improving organizational conditions (22%). No relevant gender differences could be demonstrated for any of the proposed measures (*d* < 0.20).

### Associations Between Work-, Person-, and Health-Related Characteristics With Retirement

The correlation analyses examined the relationship between work-, person-, and health-related characteristics and age with the variable early versus regular retirement for both genders. The strength of the examined correlations did not differ between the genders. The work-related characteristics do not show any statistical significant correlation with the variable retirement start date (*r* = −0.08–0.01). The effort-reward subscales correlate low with prediction of retirement (*r* = −0.31 to −0.28), i.e., low occupational effort, high reward, and low ER ratio tend to be associated with attainment of regular retirement age.

The same applies to the correlations between retirement and personal characteristics (*r* = −0.29 to −0.21) or emotional exhaustion (male: *r* = −0.35, female: *r* = −0.34): The more favorable ability to recover, overcommitment and emotional exhaustion are, the more probability there is of reaching the regular date of retirement. For age, there is a very small (*r* = −0.15) correlation for male teachers and a small (*r* = −0.21) correlation for female teachers.

Regardless of gender, the trend of increasing weekly working time for teachers is accompanied by a higher overcommitment (*r* = 0.26), reduced ability to recover (*r* = 0.26), and increased ERI (*r* = 0.23), and vice versa; while for emotional exhaustion there is only a very small correlation with weekly working time (*r* = 0.17). At the same time, increasing effort-reward ratio, overcommitment (*r* = 0.42), inability to recover (*r* = 0.49), and emotional exhaustion (*r* = 0.44) are on the rise. Emotional exhaustion is moderately correlated with overcommitment (*r* = 0.53) and inability to recovery (*r* = 0.57). And there is a strong correlation between overcommitment and inability to recover (*r* = 0.77), according to which pronounced overcommitment is associated with strong inability to recover.

Binary logistic regression analyses were calculated separately for both genders to examine the extent to which the characteristics studied contribute to reaching the regular retirement age among male and female teachers. The results of these analyses make clear that the models of the two gender groups practically do not differ. When looking at the individual (independent) characteristics, it turns out that the control variable subject profile and the working time-related characteristics hardly contribute to the explanation of reaching regular retirement age (Nagelkerke *R*^2^ < 1%). The ER model characteristics explain 8 to 13%, and the person-related characteristics 6 to 15% of the probability of reaching regular retirement age. For both gender groups emotional exhaustion (15–17%), ability to recover (11–14%), and the effort-reward ratio (12–13%) provide the highest-related explanations.

Therefore, and based on the correlation analyses, only total weekly working time, ER ratio, the person-related characteristics, and emotional exhaustion were included in the overall model, as well as age as a control variable. The propensity to overcommit and the ability to recover alone explain 11–15% and the addition of emotional exhaustion 17–21% of the variance for attainment of regular retirement age, whereby the correlation between overcommitment and ability to recover should be noted (*r* = 0.77).

The highest variance clarification could be achieved with the overall model (see Table 5). For both genders, this model is statistically significant (male/female: *χ*^2^(6) = 520.80/705.72, *p* < 0.001); however, at 25% (Nagelkerke *R*^2^ = 0.25), it shows only an acceptable goodness-of-fit between the overall model and the data (Backhaus et al., 2003), which means the independent variables explain 25% of the probability of teachers reaching regular retirement age.

**Table 5:** Binary logistic regression models of work-, person-, and health-related characteristics and covariates (age and subject profile) with reaching regular retirement of male (*n* = 2,680) and female (*n* = 3,429) full-time teachers.

Total model	Coefficient (*B*)	Standard error of *B*	Wald statistic	Value of *p*	Estimated odds ratio	Confidence interval for Exp (B)	
					Exp (B)	Lower limit	Upper limit
Male							
Working time [hours/week]	0.002	0.01	0.17	0.677	1.002	0.99	1.01
Effort-reward ratio	−0.876	0.13	45.62	<0.001^***^	0.416	0.32	0.54
Overcommitment [pts]	−0.143	0.02	40.80	<0.001^***^	0.867	0.83	0.91
Inability to recover [pts]	−0.247	0.04	17.61	<0.001^***^	0.781	0.70	0.88
Emotional exhaustion [pts]	−0.423	0.05	87.82	<0.001^***^	0.655	0.60	0.72
Age [years]	−0.034	0.00	48.12	<0.001^***^	0.966	0.96	0.98
Constant	5.441	0.42	170.45	<0.001^***^	230.784		
Female							
Working time [hours/week]	0.017	0.01	13.99	<0.001^***^	1.018	1.01	1.03
Effort-reward ratio	−0.860	0.11	580.02	<0.001^***^	0.423	0.34	0.53
Overcommitment [pts]	−0.17	0.05	11.92	0.010^**^	0.844	0.77	0.93
Inability to recover [pts]	−0.095	0.02	24.70	<0.001^***^	0.910	0.88	0.94
Emotional exhaustion [pts]	−0.454	0.04	139.62	<0.001^***^	0.635	0.59	0.68
Age [years]	−0.041	0.01	118.22	<0.001^***^	0.960	0.95	0.97
Constant	3.943	0.33	143.51	<0.001^***^	51.570		

Dependent variable: early vs. regular retirement = 0–1 coded; binary logistic regressions (method: enter), Exp (B) = expected ß. CI: confidence interval, significance (two-sided): ^***^p < 0.001, ^**^p < 0.01. Nagelkerke *R*^2^: male = 0.251, female = 0.250. For regression analyses, the problem of collinearity must be taken into account. In the analyses, overcommitment and inability to recover are correlated with *r* = 0.77. Clear collinearity is accepted for *r* > 0.90 (e.g., Tabachnick and Fidell, 2013; Harlow, 2014).

According to the percentage of accuracy in classification, only 36% of the statements of male teachers who cannot imagine reaching regular retirement age (288 out of 791) were predicted correctly. For female teachers, this concerns 54% (774 out of 1,441). In comparison, 92% of the statements made by male teachers and 81% by female teachers who stated that they intend to retire at the regular retirement start date are correctly assigned. Overall, this corresponds to a correct prediction in 76% of cases for male teachers and in 69% of cases for female teachers. All model coefficients and odds can be found in Table 5.

The main predictors for reaching the regular retirement are emotional exhaustion, ER ratio, inability to recover, and overcommitment: As the ER ratio increases and the EE increases, the chance of regular retirement is reduced by a factor of 0.416 and 0.655 for men and by a factor of 0.423 and 0.635 for women, respectively. For both genders, these correlations are weaker for the inability to recover (OR: male = 0.867, female = 0.910) and overcommitment (OR = 0.781 and 0.844, respectively).

## Discussion

Teachers regularly perform a wide range of tasks with high psychosocial and emotional demands. In doing so, the high level of autonomy in the work organization represents both a resource and a risk for the long-term health of teachers. If a good balance between work and recovery is achieved, teachers can stay in the profession for a long time. However, if an unfavorable working style leads to high levels of professional exhaustion and an inability to recover, teachers are at increased risk of stress-related mental illness and early retirement. It is therefore of paramount importance to analyze possible factors of influence in this process and to examine whether in different ways males and females endanger their own health due to particular behavior, thereby increasing the probability of early retirement.

Our study shows that the overall working time of full-time high school teachers differs only slightly ${\mathrm{(\eta}}_{\mathrm{partial}}^{2}$ < 0.01). The only difference is that, on average, female teachers spend about 2 h a week more on teaching activities than male teachers ${\mathrm{(\eta}}_{\mathrm{partial}}^{2}$ = 0.016, small effect). It is noticeable that the extent of emotional exhaustion does not differ between the genders ${\mathrm{(\eta}}_{\mathrm{partial}}^{2}$ < 0.01). Previous studies came to contradictory results (e.g., Bakker et al., 2002; Bekker et al., 2005; Rupert and Morgan, 2005). In some studies, female teachers have reported higher levels of emotional exhaustion than male teachers (Van Dick and Wagner, 2001; Wang et al., 2015; Arvidsson et al., 2016). A frequent justification for this has been the assumed greater overall burden on women due to increased family obligations and work-family conflicts (Gyllensten and Palmer, 2005). Since this study only looked at full-time teachers and noticeably few female teachers cared for children in their own household (21%), the lack of gender differences seems plausible. In addition, family responsibilities are now shared more fairly between men and women than they were 20 years ago.

It should be noted that in total around one-quarter of male teachers (23%) and one-third of female teachers (31%) have high levels of emotional exhaustion. This is a disturbing finding since it is evident that high exhaustion values are an important risk factor in deciding on early retirement or for moving to other professions (Alarcon, 2011; Van Droogenbroeck and Spruyt, 2014). Moreover, emotional exhaustion is closely linked to both work satisfaction (Klusmann et al., 2008; Skaalvik and Skaalvik, 2010) and student achievement (Klusmann et al., 2016).

In terms of personal traits, the results show significant differences in self-harming behavior between male and female teachers. For example, significantly more female teachers (35%) than male teachers (21%) are affected by a high level of overcommitment and a high inability to recover. These teachers are at increased risk to their health in the medium and long term, especially from exhaustion. Only half of teachers (51%) have normal values for both overcommitment and ability to recover (men: 59% and women: 44%).

The regression models confirm that these personal characteristics contribute to the prediction of early retirement. Emotional exhaustion, inability to recover, overcommitment, and ER ratio are identified as important predictors for entering retirement for both genders. The results imply an increase in the probability of early retirement with increasing emotional exhaustion and inability to recover, and a high level of overcommitment. Similarly, an increase in the imbalance between effort and reward (ER ratio) increases the probability of leaving the profession early. Age effects tend to be subordinate in both genders, with the slight trend that as teachers age, they are more likely to anticipate retirement. Considerably more female teachers (42%) than male teachers (30%) predict early retirement (*d* = 261, small effect).

However, the two regression models have a low sensitivity and account for only 25% of the variation in each gender group. This means that retirement age is affected by other features not studied here. Van Droogenbroeck and Spruyt (2014), in a sample of more than 3,000 Belgian teachers, 60% of whom were in employment and 40% were already retired, identified gender, emotional exhaustion, and financial security (e.g., own property) among others as significant predictors of a retirement decision. As in this study, female Belgian teachers want to retire more than their male colleagues. Irrespective of gender, two-thirds of teachers want and make use of early retirement. Teacher turnover is a long-known phenomenon in the teaching profession, with the highest dropouts in early and late career (Grissmer and Kirby, 1997). Harris and Adams (2007) showed that the early retirement of teachers is a particular issue in comparison with other professions (including nurses and social workers).

Although there are many reasons for leaving the profession in this study, it is surprising that there is a strong match between male and female teachers: high workload is the main reason for early retirement for more than three quarters (79%) of all teachers. However, excessive work demands are not only perceived by teachers subjectively (Bauer et al., 2007; Skaalvik and Skaalvik, 2010), but also are considered the most important cause of stress (Kyriacou, 2001) and reduced wellbeing (Skaalvik and Skaalvik, 2017b) in the teaching profession. In addition, high work demands have been proven to be related to emotional exhaustion (Antoniou et al., 2006; Hakanen et al., 2006; Skaalvik and Skaalvik, 2017a; Baeriswyl et al., 2021).

Whether high work requirements ultimately become a health risk depends on the working conditions themselves and how an individual deals with these requirements. The effort-reward model (Siegrist et al., 2009) contains both explanatory approaches to the relationship between work requirements and health, including the intrinsic feature overcommitment. Both ERI (ER ratio > 1) as well as an excess tendency to overcommit were identified as predictors for early retirement. For the ER ratio, there was no gender effect, but for the overcommitment there was. It is worth noting that over a third (35%) of all teachers surveyed reported an ERI. In contrast, the share of ERI was significantly lower (22%) in a previous study among German teachers by Unterbrink et al. (2007). They also showed no gender effects in the ER ratio; however, they found an age effect. Teachers aged 45 and over reported higher ER ratios than their younger colleagues. Hinz et al. (2016), in contrast, in a recent German study, stated a slightly lower ER ratio for female teachers (0.63) than for male teachers (0.69; $\eta_{\mathrm{partial}}^{2}$ = 0.11, medium effect), without evidence of a significant age effect. Although the current gender impact study appears inconsistent. Siegrist (2017) drew attention to the high prevalence of ERI in education and showed a link between ERI, exhaustion, and depression in teachers.

Overcommitment may also increases the risk of exhaustion (Bakker et al., 2000; Wang et al., 2015). Our results show significant gender differences for overcommitment. Almost half of female teachers (47%) and at least one-third (33%) of male teachers are excessively likely to overcommit. While the direct health effect of overcommitment has been demonstrated robustly, it has not yet been fully clarified whether overcommitment further moderates the relationship between effort and reward (Siegrist and Li, 2016).

The second model looked at coping patterns, the inability of recovery, and it also showed that male and female teachers cope differently with their professional needs. For example, insufficient recovery in our sample was significantly more common among female (47%) than among male teachers (38%; $\eta_{\mathrm{partial}}^{2}$ = 0.033, small effect). The argument that family responsibilities could lead to an inability for recovery is not true in our sample as a justification for gender differences, because only one in five female teachers looked after children in their own household. But it may be that female teachers find it harder to mentally detach from work content. Some studies have shown that people who tend to overcommit have a lower ability to switch off from work (Feldt et al., 2013; Wendsche and Lohmann-Haislah, 2017).

Mental detachment is an essential condition for recovery (Sonnentag and Fritz, 2015). This seems to be a particular problem in the teaching profession. Varol et al. (2021) showed, on the basis of a representative survey of German employees, that teachers report difficulties in switching off from work mentally twice as often compared to other professions (42% vs. 21%). Emotional requirements as well as time and performance pressure were the main causes of this. Overall, teachers were the second most frequently (23%) affected by recovery problems after managers (Schulz et al., 2020).

For teachers, recovery after work is particularly important, as their opportunities for rest at work are insufficient (Geurts, 2014). In addition, unfavorable working hours in the evening and the weekends hinder necessary recovery processes due to consistent physiological activation (Van der Hulst, 2003; Sonnentag and Fritz, 2015) and may stimulate rumination. Ruminating, which females have a stronger propensity for than males (Jose and Brown, 2008; Hyde, 2014), can continue the process of not being able to switch off. In a recent meta-analysis, Karabinski et al. (2021) showed that a variety of interventions can effectively support detaching from work, especially when the programs use either boundary management strategies, emotional regulation techniques, or strategies to improve sleep quality as key elements. Interventions with higher intensity and longer duration achieved the greatest success. Older participants and those with health impairments benefited more from the programs.

In summary, the present study shows that for full-time high school teachers, the work-related characteristics are not differ between the genders ${\mathrm{(\eta}}_{\mathrm{partial}}^{2}$ < 0.01). Even for emotional exhaustion, the differences between the two gender groups are not relevant. On the other hand, the personal characteristics of overcommitment and inability to recover are significantly less favorable for female teachers than for male teachers. As both behavioral characteristics are considered ineffective coping strategies and they further increase the stress caused by working conditions, there is evidence of more self-harming behavior among female teachers than among their male counterparts. The assumption is supported by the significantly more frequent perception of females that they cannot remain in the profession until the regular retirement age.

The originality of the study is that for the first time, data on working time, work load, and health are reported with a large and representative sample of full-time high school teachers for the whole of Germany, taking into account significant influence factors. The composition of the sample corresponds to the characteristics of gender and age of the German high school teaching population. These represent a large professional group, which throughout Germany comprises approximately 42% men and 58% women, thus allowing good comparability for the consequences of gender-specific, occupational stress (Travers and Cooper, 1991).

Another feature of this study is the relatively homogeneous sample, which only takes into account full-time upper-level high school teachers for whom the share of teaching dominates; teachers in management positions (e.g., school directors) and officials (e.g., staff councils) were consistently excluded. Both genders have comparable working conditions, which is considered a crucial prerequisite to detect gender effects (Schaufeli et al., 2001). Previous research has often looked at inhomogeneous samples and reported gender differences with no indication of effect sizes (Bauer et al., 2006; Unterbrink et al., 2007; Nübling et al., 2011; Hinz et al., 2016).

From a methodological point of view, the study is also based on differentiated working time records with 12 categories of activities over 4 weeks (online protocol). Even if this period represents only an average workload from the school year, this method of collection provides a reliable basis for determining average weekly working time (Felsing et al., 2019b). In addition, answers relating to early retirement and possible measures to achieve normal retirement age were evaluated with great effort for all 6,109 teachers and categorized according to self-developed categories. As teachers are considered experts in this context, a differentiated picture of their occupational and health sources and resources could be generated.

There are also limitations to consider when interpreting the results in this study. The data were collected as a cross-sectional study, so that it is not possible to tell causal links between the characteristics examined and the projected retirement age of the teachers. Since participation in the investigation was voluntary, it is also a convenience sample in which selection effects and a healthy worker effect cannot be excluded. As a result, health risks may have been underestimated.

Another limitation concerns data collection: since the variables were captured by self-information, known bias due to social desirability, response tendencies and memory deficits cannot be excluded. Furthermore, the probability of retirement was collected only as a single item. As the focus on this issue is on content validity, this method of survey is appropriate (Fisher et al., 2016b). According to de Boer et al. (2004), a single global issue of validity and reliability does not have to have significant disadvantages over larger sets of questions.

With regard to the regression analyses used, it should be noted that ultimately both gender and the confounders adjusted in the statistical analyses could be partially related to early retirement. In order to achieve more clarity about the influence of the variables, alternative analyses, such as a propensity score matching analyses, could be applied in the future.

## Conclusion

The study supports the known findings that teachers need more support to stay healthy. This is the most important prerequisite for dealing with the demanding work requirements, to remain efficient and to provide good teaching. The sample in this study provides a solid basis to derive proportionate and behavioral prevention measures. The key is to identify health risks at an early stage and to influence labor and health resources in such a way as to counteract widespread premature retirement among teachers.

In order to protect against overcommitment and health problems, measures are needed which focus on reducing work requirements and developing social support work environments, while at the same time focusing on individual improvement of coping strategies in dealing with the high workloads and emotional interactions. The teachers in our study themselves propose reducing the class size and the number of compulsory hours, reducing additional tasks, bureaucratic structures and administrative burdens, and above all improving the organizational and working conditions in schools. This includes providing high-quality teaching materials and creating adequate retreats at school, as well as a value-added management style and team-oriented approach among all employees. Teachers also want more realistic curricula and more time to maintain relationships with students. In addition, in the teaching profession, there is a lack of well-founded and proven human resources development strategies which maintain and promote the employability and health of teachers until their regular retirement. It would be advisable to offer preventive medical care on a regular basis, covering early indicators of health risk, such as overcommitment, inability to recover, and emotional exhaustion, and use them as a basis for individual health advice for teachers.

On an individual level, aspiring teachers should learn in their studies techniques and strategies which contribute to the regeneration and strengthening of resilience and which can be integrated into both professional and private everyday life. These includes active recreation offers, such as activities in nature, which help to switch off from work. It is also necessary for teachers to develop a healthy distance from the many requirements of the teaching profession.

In the future, it will be essential to have longitudinal studies to analyze the links between school workload and health consequences, as well as health prevention.

## Data Availability Statement

The original contributions presented in the study are included in the article materials, and further inquiries can be directed to the corresponding author.

## Ethics Statement

The studies involving human participants were reviewed and approved by the Ethics Committee of the University of Rostock (A 2018-0031). The participants provided their written informed consent to participate in this study.

## Author Contributions

RS made the funding acquisition. SK and RS designed the study, made the project administration, collected the data, made the analysis and interpretation of the data, and wrote the manuscript. Both authors contributed to the article and approved the submitted version.

## Funding

This study was funded from the German Association of Philologists (DPhV). The sponsor was not involved in data collection, analysis, interpretation, manuscript preparation, or the decision to publish.

## Conflict of Interest

The authors declare that the research was conducted in the absence of any commercial or financial relationships that could be construed as a potential conflict of interest.

## Publisher’s Note

All claims expressed in this article are solely those of the authors and do not necessarily represent those of their affiliated organizations, or those of the publisher, the editors and the reviewers. Any product that may be evaluated in this article, or claim that may be made by its manufacturer, is not guaranteed or endorsed by the publisher.
